# You cannot find what you are not looking for! detecting malaria outbreaks in Uganda: a case study

**DOI:** 10.11604/pamj.supp.2022.41.1.31191

**Published:** 2022-03-23

**Authors:** Benon Kwesiga, Phoebe Nabunya, Alex Riolexus Ario, Daniel Kadobera, Lilian Bulage, Stephen Ndugwa Kabwama, Julie Roberts Harris

**Affiliations:** 1 Uganda National Institute of Public Health, Kampala, Uganda,; 2 Ministry of Health, Kampala, Uganda,; 3 College of Health Sciences, Makerere University School of Public Health, Kampala, Uganda,; 4 US Centers for Disease Control and Prevention, Kampala, Uganda,; 5 Division of Global Health Protection, Center for Global Health, US Centers for Disease Control and Prevention, Atlanta, United States of America

**Keywords:** Case-Study, malaria, surveillance, data, analysis, outbreak, detection, Uganda

## Abstract

Malaria is the leading cause of morbidity and mortality in Uganda, with nearly half of the population becoming infected in any given year. Uganda relies on analyzing high-quality surveillance data to help detect outbreaks, determine which areas or population groups are most affected, and help target resources to where they are most needed. In March 2019, over 300 health facilities from different districts in Uganda reported substantially higher malaria cases than usual. In 13 districts, health facilities reported that the number of malaria cases was so high that they were experiencing stock outs of antimalarial drugs. Although seasonal increases in cases had been expected, districts reported that the number of cases being identified were overwhelming the capacity of the health facilities. Uganda´s National Malaria Control Division tasked a team of epidemiologists to investigate this unprecedented increase in malaria cases. National Malaria Control Division were interested in how malaria epidemiology had been changing in recent years, and whether they had missed something that would have predicted the situation they were facing in 2019. This case study describes the steps taken to conduct a descriptive analysis of routine malaria surveillance data and demonstrates how to detect malaria outbreaks using historical data. It is useful for training Field Epidemiologists and public health officers involved in analysis of surveillance data.

## How to use this case study

Case studies in applied epidemiology allow students to practice applying epidemiologic skills in the classroom to address real-world public health problems. The case studies are used as a vital component of an applied epidemiology curriculum, rather than as stand-alone tools. They are ideally suited to reinforce principles and skills already covered in a lecture or in background reading. This case study has a facilitator guide and a participant guide. Each facilitator should review the Facilitator Guide, gain familiarity with the outbreak and investigation on which the case study is based, review the epidemiologic principles being taught, and think of examples in the facilitator´s own experience to further illustrate the points. Ideally, participants receive the case study one part at a time during the case study session. However, if the case study is distributed in whole, participants should be asked not to look ahead. During the case study session, one or two instructors facilitate the case study for 8 to 20 students in a classroom or conference room. The facilitator should hand out Part I and direct a participant to read one paragraph out loud, then progressing around the room and giving each participant a chance to read. Reading out loud and in turns has two advantages. First, all participants engage in the process and overcome any inhibitions by having her/his voice heard. Second, it keeps all participants progressing through the case study at the same speed. After a participant reads a question, the facilitator will direct participants to answer the question by perform calculations, construct graphs, or engage in a discussion of the answer. Sometimes, the facilitator can split the class to play different roles or take different sides in answering the question. As a result, participants learn from each other, not just from the facilitator. After the questions have been answered, the facilitator hands out the next part. At the end of the case study, the facilitator should direct a participant to read the objectives once again on page 1 to review and ensure that the objectives have been met.

**Target audience:** trainees in Field Epidemiology and Laboratory Training Programs (FELTPs), public health students, public health workers who may participate in routine analysis of surveillance data, and others who are interested in this topic.

**Prerequisites:** for this case study, participants should have received instruction or conducted readings in: *Public health surveillance and analysis of surveillance data*; *Using disease thresholds to detect outbreaks*.

**Materials needed:** flip charts, marker set: one per 8-10 participants. Laptop computers: with Microsoft Office Suite pre-installed. Projector, projection screen (or substitutes), and LASER pointer.

**Level of case study:** frontline, intermediate, and advanced (e.g., Intermediate, i.e., participants should have background in analyzing public health surveillance data).

**Time required:** *Provide expected duration* (e.g., approximately 3 hours)

**Language**: English

## Case study material

- Download the case study student guide (PDF - 0.99 MB)
- Request the case study facilitator guide

## References

[1] President´s Malaria Initiative (2019). Uganda - Malaria Operational Plan FY 2019.

[2] Ssempiira J, Nambuusi B, Kissa J, Agaba B, Makumbi F, Kasasa S (2017). Geostatistical modelling of malaria indicator survey data to assess the effects of interventions on the geographical distribution of malaria prevalence in children less than 5 years in Uganda. PLoS One.

[3] Muhaise H (2017). Electronic health information systems critical implementation issues (E-HMIS): District Health Information Software version. 2 in the greater Bushenyi, Uganda. American Scientific Research Journal for Engineering, Technology, and Sciences.

[4] QGIS (2021). Quantum Geographical Information System [Internet].

[5] Dato V, Wagner MM, Fapohunda A (2004). How outbreaks of infectious disease are detected: a review of surveillance systems and outbreaks. Public health reports (Washington DC: 1974).

[6] World Health Organization (2004). Field guide for malaria epidemic assessment and reporting [Internet].

